# Quantitative pupillometry as a sensitive tool for detecting tumor-related mass effect in the posterior fossa: a prospective feasibility study comparing metastatic versus skull base lesions

**DOI:** 10.1007/s11060-025-05363-9

**Published:** 2025-12-08

**Authors:** Martin Grutza, Dorothea Mitschang, Viktoriya Sydorenko, Martin Dugas, Sandro M. Krieg, Pavlina Lenga

**Affiliations:** 1 Department of Neurosurgery, Heidelberg University Hospital, Im Neuenheimer Feld 400, 69120 Heidelberg, Germany; 2 Medical Faculty of Heidelberg University, Heidelberg, Germany; 3 Institute of Medical Informatics, Heidelberg University Hospital, Heidelberg, Germany

**Keywords:** Quantitative pupillometry, Posterior fossa lesions, Mass effect, Hydrocephalus

## Abstract

**Objectives:**

Early and precise assessment of tumor-related mass effect is critical for surgical decision-making in patients with posterior fossa tumors, where even subtle elevations in intracranial pressure (ICP) may rapidly precipitate neurological decline. While quantitative pupillometry (QP) has demonstrated robust predictive capability for ICP-related deterioration in traumatic brain injury, its clinical utility for assessing neuro-oncological mass effects remains unexplored. This study aims to determine whether QP can reliably detect subtle neurological dysfunction related to mass effect in posterior fossa tumors, distinguishing specifically between metastatic and benign skull base lesions.

**Methods:**

We prospectively analyzed 58 adults (mean age 55.6 ± 13.8 years, 60.3% female) who underwent microsurgical resection of posterior fossa tumors from January to May 2025. Pupillary function was evaluated pre- and postoperatively using the automated NPi 200^®^ Pupillometer. Pupillary parameters included the Neurological Pupil Index (Npi), constriction and dilation velocities, and latency. Clinical and radiological data, including tumor volume, midline shift, and Evans Index, were correlated with pupillometry metrics. Patients were categorized into metastatic (*n* = 21) and benign skull base (*n* = 37) tumor groups for subgroup analysis.

**Results:**

Benign tumors (55.2%) predominated, with skull base tumors significantly more often associated with cranial nerve deficits compared to metastases (*p* = 0.0023). Baseline pupillometric values appeared generally within normal range (median NPi = 4.4). However, significant postoperative improvements in pupillary parameters, including constriction velocity and NPi, occurred exclusively in skull base tumors. Preoperatively, NPi negatively correlated with tumor volume (ρ=–0.72, *p* = 0.008), while constriction velocity positively correlated with tumor volume (ρ = 0.73, *p* = 0.007) and midline shift (ρ = 0.60, *p* = 0.040). Latency correlated significantly with ventricular enlargement (Evans Index; ρ = 0.58, *p* = 0.046). Multivariate analysis confirmed tumor volume as an independent predictor of impaired NPi (β=–0.021, *p* = 0.015). Furthermore, in an exploratory ROC analysis among patients with skull base tumours, a preoperative NPi < 4.0 was associated with meaningful postoperative pupillary recovery (AUC = 0.81, 95% CI: 0.64–0.94) in this cohort.

**Conclusions:**

Quantitative pupillometry, particularly the NPi and constriction velocities, sensitively identifies early neurological impairment from posterior fossa tumour–related mass effect. Moreover, lower preoperative NPi values were associated with postoperative neurological improvement, suggesting that pupillometry may have potential as a bedside tool to support surgical decision-making; however, these exploratory findings require validation in larger cohorts before firm predictive claims can be made.”

**Supplementary Information:**

The online version contains supplementary material available at 10.1007/s11060-025-05363-9.

## Introduction

Posterior fossa tumors present significant neurosurgical challenges due to their anatomical proximity to critical brainstem and cranial nerve structures, as well as their propensity for rapid neurological deterioration from even minor volumetric or intracranial pressure (ICP) changes. The confined anatomy of the posterior fossa renders lesions particularly susceptible to mechanical displacement and rapid compression of vital autonomic and sensorimotor pathways within the midbrain, pons, and medulla oblongata. Conventional neurological assessments often fail to detect subtle neurological compromise prior to overt clinical deterioration, particularly in sedated or critically ill patients [1–4]. Quantitative pupillometry (QP) has emerged as an objective bedside tool for real-time neurological assessment, offering reproducible measurements of pupillary reactivity [5, 6]. The NPi, a composite measure of pupillary reactivity ranging from 0 (non-reactive) to 5 (maximally reactive), has demonstrated predictive value in conditions characterized by intracranial hypertension or impending herniation, including traumatic brain injury and large hemispheric stroke [7–10].

In neuro-oncology, however, the clinical implications of QP remain insufficiently explored, particularly regarding posterior fossa tumors, which differ fundamentally in pathophysiology. Metastatic lesions typically exhibit infiltrative growth with associated vasogenic edema, whereas skull base tumors (e.g., vestibular schwannomas, meningiomas) predominantly induce symptoms through mechanical compression without direct infiltration [11–13]. Such distinct pathological mechanisms likely produce unique pupillometric signatures, yet comparative studies are currently lacking. Furthermore, MRI-based volumetric assessments (e.g., midline shift, fourth ventricular distortion, Evans index) have limited correlation with real-time brainstem function as measured by pupillometry [14, 15]. QP thus has potential as an adjunctive tool bridging structural imaging and bedside neurological monitoring.

To address these gaps, we conducted a prospective observational study evaluating baseline and early postoperative pupillometry parameters in patients with posterior fossa tumors. We hypothesized that metastatic tumors, given their infiltrative nature, would produce greater pupillary dysfunction at smaller tumor volumes, whereas benign skull base lesions would primarily induce reversible pupillary impairment via mechanical displacement, rapidly resolving postoperatively. Therefore, the aim of this prospective feasibility study was fourfold: (i) to characterise pre- and early postoperative quantitative pupillometry in adults with posterior fossa tumours; (ii) to relate pupillary parameters to radiological markers of mass effect; (iii) to compare pupillary trajectories between metastatic and benign skull base lesions; and (iv) to explore whether quantitative pupillometry parameters are associated with postoperative changes in pupillary function and may inform future predictive approaches to guide surgical decision-making.

## Methods

### Data collection, inclusion and exclusion criteria

All clinical and imaging data for this prospective analysis were sourced from our institutional database focused on intracranial pathologies. For this specific investigation, we consecutively enrolled adult patients (≥ 18 years) who underwent surgical resection of posterior fossa tumors at our neurosurgical department between January 2025 and May 2025. This study was conducted in accordance with the Declaration of Helsinki (2013). The protocol was reviewed and approved by the institutional ethics committee (Approval No. S-788/2021), which granted a waiver of informed consent because QP is part of routine institutional clinical care and only de-identified data were analyzed. Patients were eligible if they had radiologically confirmed tumors located in the posterior cranial fossa and underwent microsurgical tumor resection. All surgical procedures adhered strictly to standardized operative protocols. A mandatory requirement for inclusion was the availability of pre- and postoperative pupillometric assessments. Patients lacking these measurements or with incomplete clinical/imaging records were excluded. Baseline demographics, clinical variables, tumor entities (classified as metastatic or skull base tumors), and operative details were meticulously extracted from electronic patient records and MRI studies. During the study period, QP was part of routine perioperative neurological monitoring and the NPi values were documented in the medical record; treating clinicians were therefore not formally blinded to the results. However, there was no predefined algorithm or threshold by which QP findings were used to determine surgical indication, timing or approach, and all patients included in the study had an established indication for posterior fossa tumour resection based on standard clinical and radiological criteria. Systemic comorbidities (e.g. hypertension, diabetes mellitus) and medication histories were documented as part of routine perioperative care but were not pre-specified as covariates in this exploratory QP analysis. Detailed ophthalmologic diagnoses and formal anisocoria thresholds were not systematically coded beyond the standard neurosurgical neurological examination.

All adults undergoing posterior fossa tumour resection during the study period were screened for eligibility. Quantitative pupillometry and MRI were performed as part of routine perioperative care. Only patients with interpretable pre- and early postoperative pupillometry and complete imaging datasets were included in the final analysis. Missing measurements were largely attributable to logistical or technical reasons (e.g. temporary unavailability of the pupillometer or omission of early postoperative measurements), rather than to clinical exclusion criteria. Tumour entities were first classified histopathologically as malignant (metastases, sarcomas, solitary fibrous tumours) or benign (schwannomas, meningiomas, epidermoids, haemangioblastomas) according to WHO criteria.

### Pupillometry assessments

Automated quantitative pupillometry was performed using the handheld infrared NPi-200^®^ Pupillometer (NeurOptics, Laguna Hills, CA, USA). This validated device records pupil dynamics automatically over a three-second interval, calculating the NPi. The NPi is an algorithm-generated scale from 0 to 5, where values ≥ 4.0 are considered normal, and scores below 3.0–4.0 indicate progressively more significant pupillary reflex abnormalities, potentially reflecting neurological dysfunction [16, 17]. The device captures additional parameters, including pupil size, latency, constriction velocity, and dilation velocity, allowing comprehensive quantitative assessment of pupillary function. At each time point, quantitative pupillometry was performed sequentially for both eyes, and NPi and all dynamic parameters were recorded separately for the right and left eye. For all inferential analyses (correlations, GEE models, and ROC analysis), the unit of analysis was the individual eye; right- and left-eye values were analysed separately, and patient ID was used as the clustering variable to account for within-patient correlation between eyes. No averaging of bilateral NPi values was performed for the main analyses.

Examinations were performed at the bedside in the intensive care unit or neurosurgical ward with patients in a supine or semi-recumbent position. Measurements were obtained in a dimly lit environment, and care was taken to avoid direct ambient light entering the eye. The device was positioned according to the manufacturer’s instructions, and its sealed rubber eyecup and internal infrared light source standardise the stimulus and recording conditions. No mydriatic or miotic eye drops were administered within at least 24 h prior to pupillometry, and contact lenses or spectacles were removed if they interfered with the measurement. For each patient, measurements were obtained once within 24 h preoperatively and once in the early postoperative phase, between 24 and 72 h after surgery. The postoperative assessment was scheduled as soon as feasible after emergence from general anaesthesia, when patients were awake or easily arousable and hemodynamically stable. Continuous intravenous sedative infusions were not maintained at the time of pupillometry; postoperative analgesia followed institutional, predominantly opioid-sparing protocols. This time window was chosen to capture early postoperative changes in brainstem function while minimising the influence of immediate anaesthetic recovery and ensuring feasibility within routine clinical workflows. Pupillometric evaluations were conducted once within 24 h preoperatively and once between 24 and 72 h postoperatively to detect immediate neurological changes related to surgical decompression. Given the low inter-observer variability reported in previous studies [17], repeated measurements at each time point were not conducted.

#### Tumor classification

Pre- and early postoperative MRI were analysed for tumour volume, fourth-ventricle volume, anteroposterior (AP) displacement of the fourth ventricle, midline shift, diameter of the lateral ventricles and Evans Index. For comparative analyses, tumours were classified into (i) metastatic lesions (including one cerebellar lymphoma) and (ii) non-metastatic skull base tumours. The latter group comprised meningiomas, vestibular schwannomas and other lesions located at the posterior fossa skull base (e.g. epidermoids, haemangioblastomas, cavernous malformations, chondrosarcomas). This anatomically based dichotomisation was defined a priori to reflect differences in growth dynamics and mass-effect patterns rather than histopathology alone. In addition, an exploratory histology-stratified comparison between meningiomas and vestibular schwannomas is provided in Supplementary Table S9.

#### Tumor topology

In addition to histopathological diagnosis, tumour topology was characterised on pre-operative MRI. For each lesion we recorded (i) the dominant brainstem level of compression (midbrain, pons, medulla, or none), (ii) whether the primary cerebellar involvement was hemispheric, vermian, or both, and (iii) the presence of fourth-ventricle distortion. Schwannomas were subclassified according to the involved cranial nerve and typical location (vestibular/cochlear [cerebellopontine angle], trigeminal [Meckel’s cave], facial, lower-cranial-nerve/vagal [jugular foramen region]). Meningiomas were subclassified by dural attachment as petroclival, cerebellopontine angle (CPA), tentorial, foramen magnum, or other skull-base locations. For the main comparative analyses of pupillary dynamics, lesions were additionally grouped according to anatomical growth pattern into metastases versus skull base tumours. The metastasis subgroup comprised intra-axial posterior fossa metastases and one primary CNS lymphoma, whereas the skull base subgroup included extra-axial lesions centred at the posterior fossa skull base (schwannomas, skull-base meningiomas, epidermoids, haemangioblastomas with skull-base extension, and malignant skull-base primaries). Thus, the histological and anatomical groupings partially overlap and are not intended to be mutually exclusive.

### Radiological assessments and volumetric measurements

Preoperative magnetic resonance imaging (MRI) was performed routinely in all patients using a standardized protocol. All radiological analyses and volumetric measurements were independently evaluated by two experienced neurosurgeons (P.L. and D.M.) with substantial expertise in posterior fossa pathologies. Any disagreements were resolved by consensus review. Inter-observer reliability was quantified using Cohen’s kappa coefficient, consistently exceeding 0.80, thus confirming excellent agreement [18].

### Quantitative imaging measurements

#### Tumor volume

Tumor volumes were manually segmented on contrast-enhanced axial MRI sequences using iPlan Net Cranial 3.0 software (Brainlab AG, Munich, Germany). Segmentation was performed independently by two neurosurgeons (P.L., 6 years’ experience; D.M., 3 years’ experience).

#### Fourth ventricle volume

Fourth ventricular volumes were similarly segmented manually on high-resolution axial MRI sequences. Normal adult volumes typically range from 0.9 to 1.6 mL; deviations suggest mass effect or obstruction.

#### Twinning line measurement (Anteroposterior displacement)

Zimmerman et al. previously described the Twinning line ratio, quantifying the anteroposterior (A–P) displacement of the fourth ventricle [19]. This ratio is derived by measuring the distance from a fixed anatomical landmark (tuberculum sellae) to the center of the fourth ventricle, divided by the distance from tuberculum sellae to the torcula. Normal ratios approximate 0.50 (range 0.47–0.53); significant deviations suggest tumor-induced A–P displacement.

#### Midline shift (Lateral displacement)

Midline shifts were assessed on axial MRI by drawing the anatomical midline (defined by falx cerebri or clival landmarks) and measuring lateral displacement of the fourth ventricle center.

#### Evans index

The Evans Index (the ratio of maximal frontal horn ventricular width to maximal internal skull diameter) was calculated using standard axial MR images. Values above 0.3 typically indicate ventricular enlargement or early hydrocephalus [20].

**Fig. 1:**
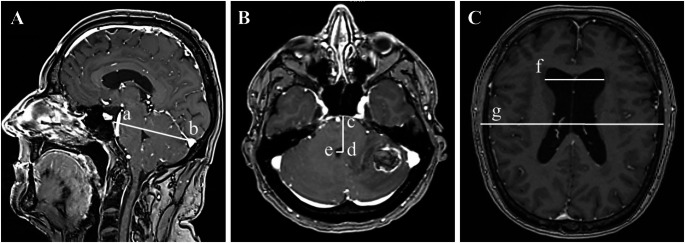
Example of Radiological Measurements in a Patient with Metastasis of the Posterior Fossa. Panels **A–C**: metastasis in the cerebellar hemisphere. (**A**) Sagittal measurement of the Twining line from tuberculum sellae to torcula (a-b = 7.9 cm); The distance from the tuberculum sellae to the centre of the fourth ventricle (c–d = 3.5 cm) is used to calculate the anteroposterior (AP) displacement ratio (c–d / a–b = 0.4). (**B**) Axial image showing lateral displacement of the fourth ventricle (d-e = 0.5 cm) as a measure of midline shift. (**C**) Evans Index (0.4) calculated as maximal frontal horn width (f = 4.6 cm) divided by maximal inner skull diameter (g = 12.9 cm)

All radiological measurements are presented in Fig. 1.

Radiological brainstem compression was rated on pre-operative MRI by an experienced neuroradiologist blinded to pupillometry results. Compression was considered present when the tumour visibly indented or distorted the brainstem and fourth ventricle on mid-sagittal or axial images; absence was defined as preserved brainstem contour without measurable effacement.

### Statistical analysis

This was a prospective feasibility and exploratory cohort study. Because no prior data on quantitative pupillometry in patients with chronic posterior fossa tumours were available to inform an effect-size assumption, no formal a priori sample-size calculation was performed. Instead, all consecutive eligible adults undergoing posterior fossa tumour resection between January and May 2025 at our tertiary neurosurgical centre were included. For subgroup analyses, “pre-operative neurological signs” were defined as any focal neurological deficit attributable to the posterior fossa lesion (cranial nerve deficit, motor deficit, or cerebellar symptoms). Patients were dichotomised into those with at least one sign and those without any neurological signs at presentation. Descriptive analyses included frequency counts and percentages for categorical variables and median (interquartile range, IQR) for continuous variables. The normality of data distribution was tested using the Shapiro-Wilk test. Pre- and postoperative differences in pupillometric parameters (NPi, constriction velocity, pupil size, latency etc.) were assessed using the Wilcoxon signed-rank test (paired analyses). Univariable comparisons between tumor entities (metastasis versus skull base tumors) were performed using Mann-Whitney U tests for continuous data and Fisher’s exact test for categorical data. Multivariable linear regression analyses were conducted to identify independent predictors of NPi, considering tumor volume, midline shift, and tumor entity (metastasis vs. skull base) as covariates. Correlations between quantitative pupillometry variables and radiological markers were explored using Spearman’s rank correlation coefficients (ρ). Given the large number of tests, we additionally applied a Benjamini–Hochberg false discovery rate (FDR) correction across all 288 correlation tests presented in Supplementary Tables 1–10. We report unadjusted p-values in the main correlation tables and provide FDR-adjusted q-values for correlations with *p* < 0.05 in Supplementary Table 8. These analyses are considered exploratory. Repeated-measures analyses (Generalized Estimating Equations, GEE) evaluated NPi changes pre- and postoperatively, including interaction terms (entity × time). The primary longitudinal analysis used generalized estimating equations (GEE) with patient as the unit of analysis and an exchangeable working correlation structure. To avoid inflating the effective sample size by including both eyes per patient, the left-sided NPi was prespecified as the primary pupillometry outcome in the GEE and ROC analyses; right-sided results were examined descriptively and were concordant. Patient ID was specified as the clustering variable so that within-patient correlation between pre- and postoperative measurements was accounted for. Receiver operating characteristic (ROC) curves were used in an exploratory manner to examine whether preoperative NPi could discriminate between patients with and without postoperative clinically meaningful improvement. In line with previous pupillometry studies and manufacturer guidance, which consider an NPi difference of ≥ 0.7 between eyes as clinically relevant and abnormal (Olson and Fishel, 2016; Privitera et al., 2022) we operationally defined “meaningful postoperative improvement” as an increase in NPi of ≥ 0.7 in the same eye (ΔNPi ≥ 0.7) All statistical analyses were conducted using R (version 4.2.0, R Foundation, Vienna, Austria) and SPSS (version 24.0, IBM Corp., Armonk, NY, USA). Statistical significance was defined as a two-tailed p-value < 0.05.

## Results

### Patient demographics

The study cohort consisted of 58 patients diagnosed with posterior fossa tumors, presenting a mean age of 55.6 years (SD ± 13.8). Females comprised the majority (60.3%). Malignant tumors were identified in 43.1% of cases, predominantly metastases (36.2%), while benign tumors were present in 55.2% of patients, primarily meningiomas (24.1%) and neurinomas (15.5%). Cranial nerve deficits were present in 27/58 patients (46.6%), motor weakness in 2/58 (3.4%), and cerebellar symptoms such as gait or limb ataxia and vertigo in 36/58 (62.1%).

Detailed demographic characteristics are presented comprehensively in Table 1.

**Table 1:** Demographics of 58 patients with posterior fossa tumors

	Value
**Mean Age in years (SD)**	55.6 (13.8)
**Sex (n**,** %)**	
Female	35 (60.3)
Male	23 (39.7)
**Tumor entity (n**,** %)**	
Malignant	25 (43.1)
Metastasis	21 (36.2)
Sarcoma	3 (5.2)
Solitary Fibrous Tumor WHO 3	1 (1.7)
Benign	32 (55.2)
Neurinoma WHO 1	12 (20.7)
Meningioma WHO 1	14 (24.1)
Epidermoid	4 (6.9)
Hemangioblastoma	2 (3.4)
**Neurological Deficits (n**,** %)**	
Cranial Nerve Deficit	27 (46.6)
Motor Deficit	2 (3.4)
Cerebellar Symptoms	36 (62.1)

Abbreviations: SD = Standard Deviation, WHO** =** World Health Organization

### Pupillometry and imaging data

Preoperative pupillometric assessment demonstrated bilateral median NPi values of 4.4, indicative of generally normal pupillary function across the cohort. Imaging analysis revealed a median tumor volume of 5.9 cm³ (IQR: 17.9 cm³; range: 0.3–55.7 cm³). The median fourth ventricular volume was 0.8 cm³ (IQR: 0.6 cm³), median A-P displacement of the fourth ventricle was 0.4 cm, median midline shift measured 0.2 cm, and the median Evans Index was 0.3, indicating overall normal cerebrospinal fluid (CSF) dynamics. Complete pupillometry and imaging metrics are detailed in Table 2.

**Table 2:** Imaging and pupillometry findings in the whole cohort of patients with posterior fossa tumors

	Value
**Preoperative Pupillometry Findings**	
**Right**	
NPi (IQR, Range)	4.4 (0.5, 2.5–4.7)
Size (before constriction), mm (IQR, Range)	3.8 (1.5, 2.5–4.7)
Min. Pupil Size, mm (IQR, Range)	2.7 (0.8, 1.9–3.5)
Constriction Index, Percentage (IQR, Range)	29.0 (12.0, 11.0–43.0)
Constriction Velocity, mm/s (IQR, Range)	2.2 (1.4, 0.8–3.5)
Max. Constriction Velocity, mm/s (IQR, Range)	3.1 (1.9, 1.2–5.0)
Dilatation Velocity, mm/s (IQR, Range)	0.9 (0.5, 0.3–1.6)
Latency, s (IQR, Range)	0.2 (0.1, 0.2–0.3)
**Left**	
NPi (IQR, Range)	4.4 (0.5, 2.5–4.8)
Size (before constriction), mm (IQR, Range)	3.7 (1.3, 2.4–6.4)
Min. Pupil Size, mm (IQR, Range)	2.6 (0.7, 1.9–4.4)
Constriction Index, Percentage (IQR, Range)	30.0 (12.0, 10.0–45.0)
Constriction Velocity, mm/s (IQR, Range)	2.4 (1.1, 0.5–3.4)
Max. Constriction Velocity, mm/s (IQR, Range)	3.2 (1.9, 0.9–5.2)
Dilatation Velocity, mm/s (IQR, Range)	1.0 (0.4, 0.3–1.5)
Latency, s (IQR, Range)	0.2 (0.1, 0.2–0.4)
**Postoperative Pupillometry Findings**	
**Right**	
NPi (IQR, Range)	4.4 (0.7, 1.1–4.9)
Size (before constriction), mm (IQR, Range)	3.4 (1.1, 2.3–6.3)
Min. Pupil Size, mm (IQR, Range)	2.5 (0.7, 1.7–4.6)
Constriction Index, Percentage (IQR, Range)	29.0 (11.0, 6.0–40.0)
Constriction Velocity, mm/s (IQR, Range)	1.9 (1.2, 0.4–3.8)
Max. Constriction Velocity, mm/s (IQR, Range)	2.9 (1.8, 0.7–5.4)
Dilatation Velocity, mm/s (IQR, Range)	0.9 (0.4, 0.2–1.4)
Latency, s (IQR, Range)	0.2 (0.1, 0.1–0.4)
**Left**	
NPi (IQR, Range)	4.5 (0.5, 1.3–4.9)
Size (before constriction), mm (IQR, Range)	3.5 (1.2, 2.0–5.8)
Min. Pupil Size, mm (IQR, Range)	2.4 (0.7, 1.6–4.3)
Constriction Index, Percentage (IQR, Range)	27.0 (14.0, 6.0–48.0)
Constriction Velocity, mm/s (IQR, Range)	2.1 (1.2, 0.5–3.7)
Max. Constriction Velocity, mm/s (IQR, Range)	3.2 (1.9, 0.6–5.4)
Dilatation Velocity, mm/s (IQR, Range)	0.9 (0.5, 0.2–1.4)
Latency, s (IQR, Range)	0.2 (0.1, 0.2–0.4)
**Imaging**	
Tumor Volume (cm³), median (IQR, range)	5.9 (17.9, 0.3–55.7)
4th Ventricle Volume (cm³), median (IQR, range)	0.8 (0.6, 0.2–2.1)
AP-Displacement of 4th Ventricle, median (IQR, range)	0.4 (0.1, 0.3–0.6)
Midline Shift (Lat. Displacement), median (IQR, range)	0.2 (0.4, 0.0–8.0)
Evans Index, median (IQR, range)	0.3 (0.1, 0.2–0.4)

Abbreviations: NPi = Neurological Pupil Index, IQR = Interquartile Range

## Tumor topology and phenotype detail

Among the skull-base tumours (*n* = 37), there were 9 schwannomas and 14 meningiomas. All schwannomas were vestibular schwannomas of the cerebellopontine angle (CPA–acoustic); no trigeminal or lower–cranial-nerve schwannomas were included. The 14 meningiomas were located petroclivally in 8 patients, tentorial in 2, at the CPA in 2, at the foramen magnum in 1 and in the pineal region in 1. Considering all 58 patients, 26 tumours were intra-axial lesions of the posterior fossa hemispheres, 2 arose from the fourth ventricle/vermis region, and the remaining lesions were extra-axial skull-base masses centred on the petroclival region (*n* = 10), CPA (*n* = 15, including 9 CPA–acoustic), tentorium (*n* = 2), foramen magnum (*n* = 1), clivus (*n* = 1) and pons (*n* = 1). Brainstem compression was present in 25/58 (43%) patients; radiology reports localised this predominantly to the cerebellar hemisphere/CPA region (hemisphere *n* = 14, CPA *n* = 7), with explicit pontine involvement in 3 cases and midbrain involvement in 1 case, while no lesion was coded as isolated medullary compression.

### Comparative analysis by tumor entity

Patients were subdivided based on tumor histology into metastases (*n* = 21) and skull base tumors (*n* = 37). While no significant differences were observed regarding age or sex distribution between these groups, cranial nerve deficits occurred significantly more frequently among patients with skull base tumors (62.2%) compared to patients with metastases (19.1%; *p* = 0.0023). Full comparative demographic results are summarized in Table 3. Preoperative pupillometric findings were comparable between the two tumor groups. However, postoperative analyses revealed significant differences, predominantly showing improvement among skull base tumor patients. Notably, significant postoperative improvement occurred in the right eye constriction index (*p* = 0.0079), constriction velocity (*p* = 0.0250), maximum constriction velocity (*p* = 0.0281), and left eye NPi (*p* = 0.0464), reflecting functional pupillary recovery largely restricted to the skull base tumor group. AP-displacement of the fourth ventricle did not differ significantly between metastases and skull-base tumours (metastasis median 0.39 [IQR 0.09, range 0.27–0.58] vs. skull-base 0.43 [IQR 0.06, range 0.35–0.56]; *p* = 0.067). Detailed comparative pupillometry and imaging parameters by tumor entity are presented in Table 4.

**Table 3:** Comparison of demographics between patients with skull base tumors and metastasis in the posterior fossa

Parameter	Metastasis *n* = 21	Skull Base Tumor *n* = 37	*p*-Value
**Mean Age in years (SD)**	55.7 (13.9)	55.7 (14.0)	0.0540
**Sex (%)**			
Female	12 (57.1)	24 (64.9)	0.5853
Male	9 (42.9)	13 (35.1)	0.5853
**Neurological Deficits**			
Cranial Nerve Deficit (%)	4 (19.1)	23 (62.2)	**0.0023**
Motor Deficit (%)	2 (9.5)	1 (2.7)	0.5462
Cerebellar Symptoms (%)	16 (76.2)	20 (54.1)	0.1585

Abbreviations: SD** =** Standard Deviation

**Table 4:** Comparison of pupillometry and imaging findings between patients with metastasis in the posterior fossa and skull base tumors

	Metastasis	Skull Base Tumor	*p*-Value
**Preoperative Pupillometry Findings**			
**Right**			
NPi (IQR, Range)	4.4 (0.6, 3.0–4.8)	4.4 (0.5, 2.2–4.7)	0.7260
Size (before constriction), mm (IQR, Range)	3.9 (1.5, 2.7–6.7)	3.8 (1.4, 2.5–6.5)	0.9449
Min. Pupil Size, mm (IQR, Range)	2.7 (0.9, 2.2–4.6)	2.7 (0.6, 2.0–4.5)	0.7609
Constriction Index, Percentage (IQR, Range)	27.0 (15.0, 16.0–41.0)	29.0 (10.5, 11.0–43.0)	0.6448
Constriction Velocity, mm/s (IQR, Range)	2.0 (1.5, 0.9–3.5)	2.2 (1.0, 0.8–3.3)	0.8701
Max. Constriction Velocity, mm/s (IQR, Range)	3.0 (2.2, 1.3–5.0)	3.2 (1.9, 1.2–5.7)	0.7837
Dilatation Velocity, mm/s (IQR, Range)	0.9 (0.5, 0.4–1.6)	0.9 (0.4, 0.3–1.5)	0.6181
Latency, s (IQR, Range)	0.2 (0.1, 0.2–0.3)	0.2 (0.1, 0.2–0.3)	0.9090
**Left**			
NPi (IQR, Range)	4.4 (0.6, 2.7–4.8)	4.4 (0.4, 2.7–4.8)	0.5296
Size (before constriction), mm (IQR, Range)	3.7 (1.6, 2.4–6.4)	3.8 (1.2, 2.4–6.3)	0.8517
Min. Pupil Size, mm (IQR, Range)	2.6 (0.8, 1.9–4.1)	2.5 (0.7, 2.1–4.4)	0.9777
Constriction Index, Percentage (IQR, Range)	27.0 (12.0, 11.0–44.0)	31.0 (11.5, 10.0–45.0)	0.5396
Constriction Velocity, mm/s (IQR, Range)	2.1 (1.3, 0.5–3.2)	2.4 (1.0, 0.9–3.4)	0.2586
Max. Constriction Velocity, mm/s (IQR, Range)	2.7 (2.2, 0.9–5.2)	3.5 (1.5, 1.2–4.8)	0.3660
Dilatation Velocity, mm/s (IQR, Range)	1.0 (0.4, 0.3–1.5)	0.9 (0.4, 0.4–1.5)	0.8020
Latency, s (IQR, Range)	0.3 (0.1, 0.2–0.4)	0.2 (0.1, 0.2–0.3)	0.2771
**Postoperative Pupillometry Findings**			
**Right**			
NPi (IQR, Range)	4.4 (0.7, 1.1–4.9)	4.5 (0.7, 3.3–4.8)	0.1776
Size (before constriction), mm (IQR, Range)	3.1 (1.0, 2.3–5.6)	3.5 (1.0, 2.4–6.3)	0.5376
Min. Pupil Size, mm (IQR, Range)	2.6 (0.7, 1.7–4.6)	2.4 (0.7, 1.8–4.4)	0.6174
Constriction Index, Percentage (IQR, Range)	22.0 (10.0, 6.0–40.0)	29.0 (6.5, 14.0–40.0)	**0.0079**
Constriction Velocity, mm/s (IQR, Range)	1.8 (0.6, 0.4–3.2)	2.3 (1.2, 0.8–3.8)	**0.0250**
Max. Constriction Velocity, mm/s (IQR, Range)	2.6 (1.1, 0.7–5.3)	3.5 (1.5, 1.3–5.4)	**0.0281**
Dilatation Velocity, mm/s (IQR, Range)	0.8 (0.5, 0.2–1.2)	1.0 (0.3, 0.5–1.4)	0.4431
Latency, s (IQR, Range)	0.2 (0.1, 0.1–0.4)	0.2 (0.1, 0.2–0.3)	0.2385
**Left**			
NPi (IQR, Range)	4.4 (0.8, 1.3–4.9)	4.5 (0.5, 3.5–4.8)	**0.0464**
Size (before constriction), mm (IQR, Range)	3.3 (1.1, 2.4–4.7)	3.5 (1.2, 2.0–5.8)	0.5719
Min. Pupil Size, mm (IQR, Range)	2.4 (0.8, 1.7–4.3)	2.4 (0.6, 1.6–3.8)	0.6897
Constriction Index, Percentage (IQR, Range)	25.0 (14.0, 6.0–45.0)	29.0 (10.5, 11.0–48.0)	0.1470
Constriction Velocity, mm/s (IQR, Range)	1.8 (1.2, 0.5–2.8)	2.3 (1.3, 0.5–3.7)	0.0647
Max. Constriction Velocity, mm/s (IQR, Range)	2.4 (1.9, 0.6–4.7)	3.6 (2.0, 0.7–5.4)	0.1237
Dilatation Velocity, mm/s (IQR, Range)	0.9 (0.6, 0.2–1.4)	0.9 (0.5, 0.3–1.4)	0.8130
Latency, s (IQR, Range)	0.2 (0.1, 0.2–0.4)	0.2 (0.1, 0.2–0.3)	0.1851
**Imaging**			
Tumor Volume (cm³), median (IQR, range)	5.8 (16.6, 0.5–35.5)	6.1 (15.3, 0.3–55.7)	0.8688
4th Ventricle Volume (cm³), median (IQR, range)	0.7 (0.5, 0.2–2.1)	0.8 (0.6, 0.3–2.1)	0.5138
AP-Displ. of 4th Ventricle, median (IQR, range)	0.43 (0.06, 0.35–0.56)	0.39 (0.09, 0.27–0.58)	0.067
Midline Shift (Lat. Displ.), median (IQR, range)	0.3 (0.6, 0.0–8.0)	0.2 (0.3, 0.0-0.7)	0.0741
Evans Index, median (IQR, range)	0.3 (0.1, 0.2–0.4)	0.3 (0.1, 0.2–0.4)	0.4063

Abbreviations: NPi** =** Neurological Pupil Index, IQR **=** Interquartile Range

## Subgroup analyses by entity, brainstem compression and neurological signs

In an exploratory analysis restricted to skull base meningiomas (*n* = 14) and vestibular schwannomas (*n* = 9), pre- and postoperative NPi values and changes in NPi did not differ significantly between the two histologies (all *p* ≥ 0.44), suggesting that the overall skull base tumour pattern is driven by shared anatomical and mass-effect characteristics (Supplementary Table S7). To assess more clinically oriented categories, patients were additionally stratified by radiological brainstem compression and by pre-operative neurological signs. Those with MRI-confirmed brainstem compression (*n* = 34) displayed slightly lower pre-operative NPi and constriction velocities and greater postoperative improvement compared with patients without compression (*n* = 24; Supplementary Table S9). Similarly, patients presenting with neurological signs (*n* = 48) showed somewhat more impaired baseline pupillary parameters than those without deficits (*n* = 10; Supplementary Table S10). Given the small sample sizes, these findings are presented descriptively and interpreted as exploratory.

### Correlation between pupillometry and imaging findings

Correlation analyses revealed significant associations, underscoring the interplay between pupillary function and anatomical parameters. Notably in the whole cohort, preoperative left-sided NPi negatively correlated with tumor volume (Spearman ρ=–0.54, *p* = 0.013), while constriction velocity demonstrated a positive correlation with tumor volume (ρ = 0.53, *p* = 0.015) and midline shift (ρ = 0.53, *p* = 0.017). Preoperative latency measurements of the left side pupil correlated positively with diameter of the lateral ventricles (ρ = 0.55, *p* = 0.012) and Evans Index (ρ = 0.58, *p* = 0.007). Divided by entity, left-sided preoperative NPi in patients with metastasis correlated negatively with tumor volume (ρ=-0.54, *p* = 0.013), while constriction velocity demonstrated a positive correlation with tumor volume (ρ = 0.53, *p* = 0.015) and midline shift (ρ = 0.53, *p* = 0.017). Preoperative latency measurements of the left side pupil correlated positively with diameter of the lateral ventricles (ρ = 0.55, *p* = 0.012) and Evans Index (ρ = 0.58, *p* = 0.007). In the right pupil preoperative minimal pupil size positively correlated with midline shift (ρ = 0.76, *p* = 0.004) and latency negatively correlated with A-P displacement (ρ=-0.67, *p* = 0.018).

In patients with skull base tumors in the left-sided measurements strong negative correlation between preoperative NPi and tumor volume (ρ=-0.72, *p* = 0.008) was seen, as well as strong positive correlations between constriction velocity and tumor volume (ρ = 0.73, *p* = 0.007) and midline shift (ρ = 0.60, *p* = 0.040). Latency positively correlated with Evans Index (ρ = 0.58, *p* = 0.046). Comprehensive correl/ation matrices exploring these relationships in detail are provided in Supplemental Tables 1– 10 (Supplementary Material).

Exploratory correlation analyses suggested that larger tumour volume and greater midline shift tended to be associated with lower NPi and higher constriction velocity, whereas greater ventricular size and Evans Index tended to correlate with prolonged latency, particularly in left-sided measurements. Similar patterns were observed within the metastasis and skull base subgroups (Supplementary Tables 1–10).

Because a large number of correlation tests were performed (288 in total), we applied a Benjamini–Hochberg FDR correction. After correction, none of the correlations remained statistically significant (smallest q = 0.37; Supplementary Table 8). Furthermore, in an exploratory analysis, we further compared the change in NPi between eyes ipsilateral and contralateral to the main tumour location or resection side and found no consistent or statistically significant laterality effect; postoperative trajectories were similar for both eyes (data not shown).

### Multivariate regression analysis

Multivariate regression analysis was conducted to evaluate independent predictors of preoperative NPi. Results identified tumor volume as significantly predictive of preoperative NPi impairment (β=–0.021, *p* = 0.015). Midline shift exhibited a trend towards significance (β=–0.30, *p* = 0.080). Importantly, tumor histology (metastasis vs. skull base tumors) was not independently predictive of NPi outcomes after adjustment (β=–0.07, *p* = 0.71), which is consistent with the hypothesis that anatomical factors such as tumour volume and mass effect, rather than tumour histology per se, are major contributors to pupillary dysfunction in this cohort.

### Generalized estimating equations (GEE) and ROC analysis for predicting postoperative recovery

In the GEE model, the estimated mean change in NPi from pre- to postoperative measurements was − 0.07 (95% CI − 0.32 to 0.18; *p* = 0.580) in the metastasis group and + 0.13 (95% CI 0.00 to 0.25; *p* = 0.044) in the skull base group for the right eye, and − 0.12 (95% CI − 0.32 to 0.08; *p* = 0.236) versus + 0.13 (95% CI 0.02 to 0.24; *p* = 0.018) for the left eye (Table 5). These model-based estimates underline that early postoperative improvement in NPi was confined to patients with skull base tumours, whereas metastasis patients showed no meaningful change ROC analysis was performed at the patient level in the 35 skull-base patients with complete pre- and postoperative NPi measurements, using NPI gesamt preOP as the predictor and ΔNPi ≥ 0.7 as the definition of meaningful postoperative improvement. The AUC was 0.81 (95% CI 0.64–0.94). At the cut-off NPi < 4.0, both patients who subsequently showed ΔNPi ≥ 0.7 were correctly identified, yielding a sensitivity of 100% (2/2; 95% CI 34–100%), while 28 of 33 patients without such improvement had NPi ≥ 4.0, corresponding to a specificity of 85% (95% CI 69–93%).

**Table 5:** Estimated mean change in neurological pupil index (NPi) from the GEE model by tumour entity

Tumour entity	Eye	Mean ΔNPi (post–pre)	95% CI	*p*-value*
Metastasis	Right	−0.07	−0.32 to 0.18	0.580
Metastasis	Left	−0.12	−0.32 to 0.08	0.236
Skull base	Right	+ 0.13	0.00 to 0.25	0.044
Skull base	Left	+ 0.13	0.02 to 0.24	0.018

*p-value for the within-group time effect (pre vs. post) from the GEE model

Abbreviations: NPi = Neurological Pupil index; CI = confidence interval

## Discussion

This prospective observational study evaluated the clinical utility of QP in quantifying mass effect among patients with posterior fossa tumors, comparing metastatic and benign skull base entities. Despite often subtle clinical presentations, baseline pupillary dysfunction—measured by NPi—was significantly associated with increased tumor volume and greater midline shift, indicating subclinical impairment of brainstem reflexes. Although metastases and skull base tumors shared comparable median NPi values at baseline, their clinical manifestations differed markedly, suggesting that anatomical distortion rather than tumor histology primarily governs pupillary changes. Postoperatively, patients with skull base tumors demonstrated rapid recovery of pupillary function, a response absent in metastatic lesions, likely reflecting differences in surgical decompression effectiveness and tumor infiltration. Importantly, pupillometry appeared sensitive in tracking early postoperative neurological changes, and in our exploratory ROC analysis an NPi threshold < 4.0 was associated with a higher likelihood of postoperative functional improvement in patients with skull base tumours. These findings suggest that QP may help identify patients who are more likely to benefit from decompression; however, given the observational design and small sample size, this threshold should be regarded as preliminary and requires confirmation before being used for clinical decision-making.

### Radiological measures of mass effect and pupillary dynamics

Our study provides quantitative evidence that specific radiological features indicative of mass effect, notably midline shift and anteroposterior (A-P) displacement of the brainstem, correlate significantly with alterations in pupillary light reflexes as assessed by QP. Prior research supports this finding, demonstrating a link between increasing mass effect and declining pupillary reactivity. For instance, Osman et al. reported that even minimal midline shifts of central brain structures, such as the septum pellucidum and pineal gland, corresponded with decreased NPi and constriction velocity in stroke patients, highlighting the direct impact of anatomical displacement at the midbrain level, where pupillary pathways reside [21]. Similarly, Kim et al. observed trends associating increased midbrain compression, measured via pineal gland displacement and midbrain diameter ratios, with pupillary asymmetry and impaired reactivity, despite limitations in sample size [22]. These observations align with our findings, as increased tumor volume and greater midline shift correlated inversely with NPi values and pupillary constriction velocities, reflecting subtle impairment of brainstem reflex arcs even before overt clinical signs. This mirrors neurocritical care studies, where subtle elevations in intracranial pressure and resultant midbrain distortion manifest as reductions in NPi and pupillary responsiveness, often preceding observable changes in resting pupil diameter [23, 24]. Thus, it appears that even modest mechanical displacement, especially involving the midbrain or cranial nerve III, can directly impair pupillary function, providing a sensitive early marker of worsening mass effect or incipient hydrocephalus. The substantial postoperative improvement in pupillary dynamics observed in skull base tumour patients is compatible with a largely mechanical, compression-related component of pupillary impairment, but this interpretation remains speculative. Following decompression, these tumors typically demonstrate rapid normalization of pupillary reflexes, contrasting with metastatic lesions, which rarely exhibit significant early improvement, likely due to persistent infiltration and edema. From a clinical standpoint, subtle radiographic indicators such as fourth ventricular displacement, brainstem indentation, or early cerebellar tonsillar descent should heighten awareness for potential pupillary dysfunction. Indeed, recent studies emphasize this clinical-radiological link, demonstrating strong correlations between maximal midline shift and new-onset anisocoria in neuro-intensive care patients [25]. The additional stratification by radiological brainstem compression and by pre-operative neurological signs strengthens the clinical interpretation of our data: pupillary abnormalities were most evident in patients with imaging and clinical evidence of brainstem involvement, yet subtle QP alterations were also detectable in a few neurologically intact patients. These findings highlight the potential of QP to complement, rather than replace, conventional clinical assessment.

### Pupillometric differences between metastatic and skull base tumors: clinical implications

Despite comparable median baseline NPi values (~ 3–5), reflecting largely preserved pupillary reactivity, metastatic and skull base tumors presented distinct clinical patterns consistent with their anatomical differences. Metastatic lesions typically manifested through signs of raised intracranial pressure or cerebellar dysfunction, such as headaches, nausea, and ataxia, due to rapid tumor growth and edema-induced hydrocephalus, rather than focal cranial nerve (CN) deficits [26]. Conversely, skull base tumors (meningiomas, vestibular schwannomas) predominantly presented with insidious, focal CN deficits—such as hearing loss, facial weakness, or diplopia—reflecting direct nerve encasement or compression [26]. Accordingly, metastases showed fewer CN deficits despite similar baseline NPi, indicating generalized mass effect rather than direct CN infiltration as the primary driver of subtle pupillary impairment. Interestingly, QP findings improved after surgery for skull base tumors, whereas metastasis cases showed minimal postoperative change. In patients with skull base masses, removal of the tumor often immediately relieves compression of the brainstem or cranial nerves. This can restore normal afferent/efferent function of the pupil light reflex within hours to days. Supporting evidence comes from pituitary tumor surgery: Lenga et al. demonstrated that all patients with large pituitary adenomas compressing the optic chiasm had preoperative NPi < 4 in at least one eye, and after surgical decompression the NPi rose significantly, correlating with improved visual function [27]. The study’s skull base cohort likely behaved similarly – tumor resection relieved pressure on the midbrain or CN III pathways, yielding brisker constriction and larger pupil size change post-op. This aligns with known reversibility of pupillometric CN deficits when the cause is extrinsic compression (e.g. a third-nerve palsy from an aneurysm or tumor can improve when the compression is removed). By contrast, cerebellar metastases did not show significant pupillometry improvement post-resection. One reason may be that metastases often present more acutely, and their preoperative pupillometry was near-normal. Moreover, any pupillary changes in metastases might stem from diffuse edema or axonal injury that does not resolve immediately with tumor removal. The lack of postoperative NPi change suggests that in metastases, the pathophysiology of pupillary impairment is less about focal reversible compression and more about global or irreversible factors (e.g. cytotoxic edema, ischemic stretching of brainstem). Another consideration is that skull base surgeries often directly decompress cranial nerve pathways (e.g. optic or oculomotor nerve), whereas removing a cerebellar metastasis may not drastically alter brainstem compression unless there was significant pre-op mass effect. Importantly, our findings do not imply that quantitative pupillometry, or a specific NPi threshold, should be used as a stand-alone criterion for indicating or withholding surgery. Tumour size, location, radiological characteristics and the overall clinical presentation remain the primary determinants of treatment decisions. A patient with a sizeable posterior fossa mass and normal NPi may still require urgent decompression if symptoms or imaging suggest impending deterioration. In this context, QP should be regarded as an adjunctive bedside tool that complements, but does not replace, clinical examination and neuroimaging. Its added value lies in providing objective, repeatable information on brainstem function—particularly in sedated or difficult-to-examine patients—and in documenting early postoperative recovery or subtle deterioration that might otherwise go unnoticed.

### Ventricular enlargement, pupillary constriction, and pupillometry as a sensitive bedside neurological marker

Ventricular enlargement, indicated by a high Evans index (>0.3), correlates significantly with reduced and dysmetric pupillary constriction, likely reflecting dorsal midbrain compression. This phenomenon parallels clinical observations in acute hydrocephalus, where dilation of supratentorial cerebrospinal fluid (CSF) spaces leads to Parinaud’s syndrome, characterized by pupillary hyporeflexia and disrupted gaze patterns due to pretectal region compression [28]. Our findings quantitatively confirm prior evidence, demonstrating that increasing ventriculomegaly slows pupillary constriction velocity, increases latency, and can produce dysmetric waveforms through impaired midbrain modulation. Supporting data from patients with normal-pressure hydrocephalus (NPH) highlight similar patterns: clinically responsive patients show improved constriction velocity post-CSF drainage, whereas non-responders do not, underscoring pupillometry’s ability to reflect physiological changes associated with ventricular size [29]. Indeed, quantitative pupillometry has repeatedly proven superior to conventional bedside neurological examinations in detecting subtle yet clinically significant neurological changes. Automated pupillometry can reliably identify incipient cranial nerve deficits, rising intracranial pressure (ICP), or worsening brainstem dysfunction well before overt clinical signs appear [30, 31]. For instance, early changes in NPi can precede observable third-nerve palsies by several hours, allowing clinicians to anticipate and respond proactively [30]. Similarly, in neurocritical care settings, falling NPi scores frequently herald imminent complications—such as herniation or cerebral edema—even when traditional clinical exams remain unchanged, thus prompting timely imaging and intervention [31]. Pupillometry’s objective, repeatable measures minimize inter-observer variability inherent in manual pupil exams, making it an ideal tool for continuous postoperative monitoring, particularly in posterior fossa tumor patients at high risk of acute deterioration. Additionally, pupillometry quantifies functional recovery, objectively documenting improvements after surgical decompression that subjective bedside exams may overlook [32]. Its noninvasive, repeatable nature makes it feasible to monitor trends over time and respond to dynamic changes in intracranial dynamics with timely interventions.

### Pupillometric thresholds and implications

A growing body of evidence has sought to define pupillometry thresholds that predict critical clinical outcomes such as the need for decompression or likelihood of recovery. Traditionally, an NPi below 3.0 is considered abnormal [33], but recent analyses suggest that slightly higher cut-offs may better forewarn of impending neurological decline. Chen et al., in a multicenter ICU study, found that an NPi < 3.9 was the optimal threshold to rule in elevated ICP – in fact, using an NPi of ~ 4 as a screening cut-off had good sensitivity and specificity for ICP ≥ 20 mmHg [17, 34]. This indicates that when the average NPi falls into the mid-3s, clinicians should be on alert for intracranial hypertension and consider preemptive measures (imaging, osmotherapy, or surgical decompression). Similarly, the pituitary tumor series effectively used NPi < 4.0 as a red flag for chiasm compression, since all patients with large tumors had at least one eye with NPi in the 2–3 range [27]. In the context of posterior fossa tumors, the study suggests that an ROC-derived threshold (NPi around 4.0) could predict which patients might require urgent posterior fossa decompression. This aligns with acute stroke findings: Al-Obaidi et al. observed that patients who went on to transtentorial herniation had NPi values drop to ~ 2.0 in the hours before herniation, whereas those who did not herniate stayed near 4.0 [34]. Thus, an NPi trending into the low- to mid-3s may be an actionable cutoff to trigger decompressive craniectomy or ventriculostomy before irreversible brainstem injury ensues. The recent ORANGE international cohort demonstrated that patients with any episode of NPi < 3 during acute brain injury had significantly worse 6-month outcomes and a higher mortality (each 10% increase in “NPi < 3” frequency carried a 42% rise in odds of poor outcome; abnormal NPi was associated with >5-fold hazard of death) [35]. This suggests that persistently low NPi is not only a trigger for acute intervention, but also a harbinger of poor prognosis if not reversible. Conversely, patients whose NPi remains ≥ 4 have a very low chance of occult elevated ICP and generally better outcomes [34]. In neuro-oncology specifically, pupillometry might help identify those needing more aggressive management: for instance, a patient with a posterior fossa metastasis and NPi drifting to 3.0–3.5 might benefit from urgent surgical debulking or CSF diversion, even if their exam has not acutely deteriorated. Overall, the literature supports tailored thresholds: NPi in the high-3s appears to be a critical zone, and crossing below 3.0 is clearly pathological. Using ROC analyses and large cohorts, clinicians are honing pupillary index cut-offs to anticipate neurological decompensation. The potential value of QP is that trends might be directly linked to patient outcomes – a promising advancement that merges bedside exam with predictive analytics. By intervening when NPi breaches a threshold (rather than waiting for frank blown pupils), we may improve survival and neurologic recovery in patients with brain tumors and other acute brain injuries [31, 35].

#### Pharmacologic confounders

The potential influence of sedatives, opioids and other perioperative medications on pupillary dynamics warrants consideration. Experimental and clinical data indicate that such agents clearly affect pupil size and constriction amplitude, whereas the composite NPi is comparatively less susceptible to these effects at commonly used doses (Jahns et al., 2019). In our cohort, postoperative pupillometry was performed only after patients had emerged from general anaesthesia and in the absence of ongoing continuous sedative infusions; postoperative analgesia relied on standard opioid-sparing regimens. While we did not quantify cumulative drug exposure, any residual pharmacologic effects are likely non-differential between tumour entities and would therefore tend to attenuate rather than exaggerate the observed associations between NPi and radiological markers of mass effect.

### Limitations

This study has several limitations. First, the relatively small cohort size (*n* = 58) and observational design limit generalizability and the ability to firmly establish causality. As a single-centre feasibility study included a relatively small number of patients over a short predefined recruitment period and was not based on a formal a priori power calculation. The findings should therefore be interpreted as exploratory and hypothesis-generating and require confirmation in larger multicentre cohorts. Second, heterogeneity in tumor histology, volume, and anatomical location could introduce variability in pupillometry measurements, though subgroup analyses partially mitigated this issue. In addition, only patients with complete pre- and postoperative pupillometry and imaging data were included. As missing measurements were mainly due to logistical and technical constraints, a selection bias towards patients in whom full monitoring could be performed cannot be excluded, and the results may not be fully generalisable to all patients with posterior fossa tumours. detailed cranial nerve and cerebellar signs were not recorded in a standardised fashion at each time point, and postoperative symptom evolution was derived from routine narrative documentation rather than a structured scale. As a result, our ability to correlate quantitative pupillometry changes with specific symptom trajectories (e.g. nystagmus, diplopia, dysmetria) was limited. Third, postoperative follow-up was relatively short, restricting evaluation of long-term pupillary recovery and neurological outcomes. Postoperative pupillometry was obtained within a relatively broad time window of 24–72 h after surgery, and only a single postoperative measurement per patient was analysed. This pragmatic approach reflects integration of pupillometry into routine clinical workflows but may introduce variability due to differences in the exact measurement time and evolving postoperative edema. Furthermore, we did not systematically quantify exposure to sedatives and analgesics, which may still have influenced pupillary dynamics despite performing measurements after emergence from anaesthesia. These factors may have attenuated true associations and underline the need for future studies with more standardised timing, detailed pharmacological documentation and serial pupillometry to better characterise postoperative trajectories. Fourth, we performed a large number of exploratory correlation analyses between pupillometry and radiological variables. After FDR correction across all tests, none of these correlations remained statistically significant, and they should therefore be interpreted as hypothesis-generating rather than confirmatory. In addition, our multivariable regression models were necessarily parsimonious due to the limited sample size and did not include potential confounders such as age, detailed tumour location or precise pharmacological exposure, which may have influenced the observed associations. Furthermore, our interpretation that differences between skull base tumours and metastases reflect predominantly mechanical compression versus more infiltrative or diffuse injury is speculative and based on indirect evidence; the current study cannot prove these mechanistic pathways. Our definition of “meaningful improvement” as ΔNPi ≥ 0.7 was based on prior work using a 0.7 NPi differential as an abnormal threshold and has not been independently validated as a minimal clinically important difference in patients with posterior fossa tumours. Although we explored an NPi threshold associated with postoperative improvement, the study was neither designed nor powered to develop or validate a clinical decision rule. QP in its current form should therefore not be used as a stand-alone triage or treatment tool, but rather as an adjunct to conventional clinical and radiological assessment. Moreover, although QP was not incorporated into a formal decision rule and did not determine whether patients were offered surgery, treating clinicians were aware of the measurements as part of routine care, and we cannot exclude that QP findings may have influenced clinical judgement in individual cases.

Finally, although pupillometry reliably detected subtle neurological changes, its sensitivity and specificity require validation in larger, prospective cohorts before routine clinical implementation.

#### Future directions

From a clinical perspective, the most compelling use-case for QP is the detection of early or subclinical deterioration, particularly in patients who appear neurologically intact on standard examination. In our cohort, nearly half of the patients had cranial nerve deficits and over 60% had cerebellar symptoms at baseline, but a small subgroup without focal signs still demonstrated postoperative improvements in NPi. This suggests that QP may capture subtle brainstem dysfunction that is not yet evident on routine examination. Nevertheless, because detailed cranial nerve and cerebellar signs were not collected using a structured scale and postoperative symptom evolution was documented only in narrative notes, we were not able to robustly correlate QP trajectories with specific signs such as nystagmus, diplopia or dysmetria. Beyond the perioperative setting, it is conceivable that serial QP during conservative follow-up of slowly growing skull-base meningiomas could detect progressive mass effect before overt clinical deterioration; however, our study did not include longitudinal measurements and cannot address tumour growth prediction. Prospective long-term studies will be required to determine whether changes in pupillary dynamics can meaningfully prognosticate tumour progression in this context.

## Conclusion

QP effectively quantifies subtle neurological compromise due to mass effect in posterior fossa tumors, correlating significantly with imaging markers such as tumor volume, midline shift, and ventricular enlargement. Importantly, the different postoperative trajectories observed in metastatic versus skull base tumours are compatible with distinct underlying pathophysiological mechanisms. Our data support the hypothesis that mass effect plays a key role in baseline pupillary impairment, and that this component may be more readily reversible after decompression in skull base lesions than in metastases. However, this interpretation is exploratory and cannot establish causality. Overall, pupillometry emerges as a promising bedside monitoring tool that may help detect subclinical deterioration and document early postoperative recovery, but its prognostic performance must be validated in larger, independent cohorts.

## Supplementary Information

Below is the link to the electronic supplementary material.

Supplementary Material 1

## Data Availability

The datasets generated during and/or analyzed during the current study are available from the corresponding author on reasonable request.

## References

[1] Formentin C, Joaquim AF, Ghizoni E (2023) Posterior fossa tumors in children: current insights. Eur J Pediatr 182(11):4833–4850 37679511 10.1007/s00431-023-05189-5

[2] Formentin C et al (2024) Anatomy of the posterior fossa: a comprehensive description for pediatric brain tumors. Childs Nerv Syst 40(3):613–624 37999790 10.1007/s00381-023-06220-8

[3] Radu OM et al (2024) Outcomes and complications of posterior fossa surgery in sitting versus Park-Bench positions. Med (Kaunas) 60(11) 10.3390/medicina60111855 PMC11596741 39597040

[4] Turkistani AN et al (2024) Medical management for cerebellar mutism syndrome following posterior fossa surgery: A systematic review. Clin Neurol Neurosurg 242:108352 38823197 10.1016/j.clineuro.2024.108352

[5] El Ahmadieh TY et al (2021) Automated pupillometry as a triage and assessment tool in patients with traumatic brain injury. World Neurosurg 145:e163–e169 33011358 10.1016/j.wneu.2020.09.152

[6] Joseph JR et al (2020) Pupillary changes after clinically asymptomatic high-acceleration head impacts in high school football athletes. J Neurosurg 133(6):1886–1891 31770721 10.3171/2019.7.JNS191272

[7] Couret D et al (2016) Reliability of standard pupillometry practice in neurocritical care: an observational, double-blinded study. Crit Care 20:99 27072310 10.1186/s13054-016-1239-z PMC4828754

[8] Bower MM et al (2021) Quantitative pupillometry in the intensive care unit. J Intensive Care Med 36(4):383–391 31601157 10.1177/0885066619881124

[9] Zafar SF, Suarez JI (2014) Automated pupillometer for monitoring the critically ill patient: a critical appraisal. J Crit Care 29(4):599–603 24613394 10.1016/j.jcrc.2014.01.012

[10] Jahns FP et al (2019) Quantitative pupillometry for the monitoring of intracranial hypertension in patients with severe traumatic brain injury. Crit Care 23(1):155 31046817 10.1186/s13054-019-2436-3 PMC6498599

[11] San A et al (2023) Health-Related quality of life outcomes in meningioma patients based upon tumor location and treatment modality: A systematic review and Meta-Analysis. Cancers (Basel) 15(19) 10.3390/cancers15194680 PMC10571784 37835374

[12] Laigle-Donadey F et al (2005) Skull-base metastases. J Neurooncol 75(1):63–69 16215817 10.1007/s11060-004-8099-0

[13] Sunderland GJ, Jenkinson MD, Zakaria R (2016) Surgical management of posterior fossa metastases. J Neurooncol 130(3):535–542 27619980 10.1007/s11060-016-2254-2 PMC5118393

[14] Kaya I et al (2021) Edema-mass ratio based on magnetic resonance imaging as A preoperative diagnostic factor for posterior fossa metastasis. Curr Med Imaging 17(6):762–766 33655873 10.2174/1573405617666210303105006

[15] Poretti A, Meoded A, Huisman TA (2012) Neuroimaging of pediatric posterior fossa tumors including review of the literature. J Magn Reson Imaging 35(1):32–47 21989968 10.1002/jmri.22722

[16] Meeker M et al (2005) Pupil examination: validity and clinical utility of an automated pupillometer. J Neurosci Nurs 37(1):34–40 15794443

[17] Chen JW et al (2011) Pupillary reactivity as an early indicator of increased intracranial pressure: the introduction of the NPi. Surg Neurol Int 2:82 21748035 10.4103/2152-7806.82248 PMC3130361

[18] McHugh ML (2012) Interrater reliability: the kappa statistic. Biochem Med (Zagreb) 22(3):276–282 23092060 PMC3900052

[19] Zimmerman RD, Russell EJ, Leeds NE (1980) Axial CT recognition of anteroposterior displacement of fourth ventricle. AJNR Am J Neuroradiol 1(1):65–70 6779592 PMC8333437

[20] Fleuren KJR, Koehler PJ, Hoff EI (2025) The History Evans Index Eur Neurol, : pp. 1–5 10.1159/000545917 40288352

[21] Osman M et al (2019) Correlation of objective pupillometry to midline shift in acute stroke patients. J Stroke Cerebrovasc Dis 28(7):1902–1910 31031146 10.1016/j.jstrokecerebrovasdis.2019.03.055

[22] Kim ISY et al (2022) Quantitative pupillometry and radiographic markers of intracranial midline shift: A pilot study. Front Neurol 13:1046548 36561299 10.3389/fneur.2022.1046548 PMC9763295

[23] Patel S et al (2023) Diagnosis and management of elevated intracranial pressure in the emergency department. Int J Emerg Med 16(1):72 37833652 10.1186/s12245-023-00540-x PMC10571389

[24] Mazhar K et al (2021) Supratentorial intracerebral hemorrhage volume and other CT variables predict the NPi. Clin Neurol Neurosurg 200:106410 33341651 10.1016/j.clineuro.2020.106410

[25] Prescott BR et al (2022) Anisocoria and poor pupil reactivity by quantitative pupillometry in patients with intracranial pathology. Crit Care Med 50(2):e143–e153 34637415 10.1097/CCM.0000000000005272 PMC8810747

[26] Shih RY, Smirniotopoulos JG (2016) Posterior fossa tumors in adult patients. Neuroimaging Clin N Am 26(4):493–510 27712791 10.1016/j.nic.2016.06.003

[27] Lenga P et al (2024) Evaluating optic system compression in Sellar tumors: A novel application of quantitative pupillometry. Acta Neurochir (Wien) 166(1):510 39731604 10.1007/s00701-024-06401-7 PMC11682009

[28] Feroze KB, Patel BC (2025) Parinaud syndrome. StatPearls. Treasure Island (FL) 28722922

[29] Lussier BL et al (2022) Predictive value of quantitative pupillometry in patients with normal pressure hydrocephalus undergoing temporary CSF diversion. Neurol Sci 43(9):5377–5382 35750951 10.1007/s10072-022-06230-5

[30] Aoun SG et al (2019) Objective pupillometry as an adjunct to prediction and assessment for oculomotor nerve injury and recovery: potential for practical applications. World Neurosurg 121:e475–e480 30267943 10.1016/j.wneu.2018.09.140

[31] Cortes MX et al (2021) NPi as an indicator of irreversible cerebral edema: A case series. J Neurosci Nurs 53(3):145–148 33782353 10.1097/JNN.0000000000000584

[32] Olson DM et al (2016) Interrater reliability of pupillary assessments. Neurocrit Care 24(2):251–257 26381281 10.1007/s12028-015-0182-1

[33] Sandroni C, Citerio G, Taccone FS (2022) Automated pupillometry in intensive care. Intensive Care Med 48(10):1467–1470 35773500 10.1007/s00134-022-06772-4

[34] Al-Obaidi SZ et al (2019) Impact of increased intracranial pressure on pupillometry: A replication study. Crit Care Explor 1(10):e0054 32166235 10.1097/CCE.0000000000000054 PMC7063890

[35] Oddo M et al (2023) The NPi for outcome prognostication in people with acute brain injury (ORANGE): a prospective, observational, multicentre cohort study. Lancet Neurol 22(10):925–933 37652068 10.1016/S1474-4422(23)00271-5

