# Supplementary material for: Quantitative pupillometry as a sensitive tool for detecting tumor-related mass effect in the posterior fossa: a prospective feasibility study comparing metastatic versus skull base lesions
Source: J Neurooncol. 2025 Dec 8;176(1):110. doi: 10.1007/s11060-025-05363-9 (PMC12686053; doi:10.1007/s11060-025-05363-9)
Supplement: Supplementary file 1 — Supplementary Material 1 [file 11060_2025_5363_MOESM1_ESM.docx]

**Table 1.** Correlation of **Preoperative Right Sided Pupillometry** and Imaging Findings in the overall Cohort of Patients with Posterior Fossa Tumors (n=58)

|  | **NPi** | **Size** | **Min. Size** | **Constr.** | **Constr. Velocity** | **Max Constr. Velocity** | **Latency** | **Dilatation Velocity** |
| --- | --- | --- | --- | --- | --- | --- | --- | --- |
| **Tumor Volume** | 0.02 (p=0.935) | -0.15 (p=0.514) | -0.07 (p=0.767) | -0.07 (p=0.772) | 0.02 (p=0.935) | -0.03 (p=0.900) | -0.12 (p=0.613) | 0.30 (p=0.193) |
| **4th Ventricle Volume** | 0.05 (p=0.821) | 0.30 (p=0.195) | -0.36 (p=0.121) | -0.20 (p=0.409) | -0.18 (p=0.458) | 0.16 (p=0.510) | 0.05 (p=0.821) | -0.08 (p=0.724) |
| **AP-Displacement** | -0.25 (p=0.293) | 0.26 (p=0.269) | -0.32 (p=0.165) | -0.28 (p=0.226) | -0.29 (p=0.210) | 0.08 (p=0.726) | -0.24 (p=0.315) | 0.23 (p=0.337) |
| **Midline Shift** | -0.03 (p=0.885) | -0.23 (p=0.333) | 0.29 (p=0.214) | -0.19 (p=0.414) | 0.19 (p=0.430) | 0.37 (p=0.110) | -0.24 (p=0.318) | 0.01 (p=0.972) |
| **Diam. of Lat. Ventricles** | -0.24 (p=0.303) | 0.08 (p=0.753) | 0.11 (p=0.647) | 0.04 (p=0.871) | 0.09 (p=0.707) | -0.22 (p=0.358) | -0.23 (p=0.331) | 0.26 (p=0.261) |
| **Evans Index** | -0.15 (p=0.533) | -0.03 (p=0.896) | 0.03 (p=0.896) | -0.11 (p=0.648) | 0.06 (p=0.816) | -0.08 (p=0.724) | -0.35 (p=0.132) | 0.20 (p=0.398) |

**Abbreviations: NPi=** Neurological Pupil Index**, AP=** Anterior-Posterior

**Table 2.** Correlation of Preoperative **Left Sided Pupillometry** and Imaging Findings in the overall Cohort of Patients with Posterior Fossa Tumors (n=58)

|  | **NPi** | **Size** | **Min. Size** | **Constr.** | **Constr. Velocity** | **Max Constr. Velocity** | **Latency** | **Dilatation Velocity** |
| --- | --- | --- | --- | --- | --- | --- | --- | --- |
| **Tumor Volume** | **-0.54 (p=0.013)** | 0.38 (p=0.102) | -0.33 (p=0.152) | -0.03 (p=0.910) | **0.53 (p=0.015)** | -0.20 (p=0.391) | 0.33 (p=0.152) | 0.12 (p=0.613) |
| **4th Ventricle Volume** | 0.25 (p=0.283) | -0.08 (p=0.753) | 0.09 (p=0.715) | -0.26 (p=0.268) | -0.29 (p=0.220) | 0.09 (p=0.701) | -0.02 (p=0.930) | -0.18 (p=0.443) |
| **AP-Displacement** | 0.00 (p=0.995) | -0.13 (p=0.599) | 0.06 (p=0.793) | -0.24 (p=0.316) | 0.07 (p=0.769) | -0.35 (p=0.129) | -0.09 (p=0.707) | 0.30 (p=0.197) |
| **Midline Shift** | -0.33 (p=0.160) | 0.07 (p=0.785) | 0.34 (p=0.147) | -0.27 (p=0.255) | **0.53 (p=0.017)** | -0.10 (p=0.678) | 0.27 (p=0.255) | 0.05 (p=0.822) |
| **Diam. of Lat. Ventricles** | -0.09 (p=0.710) | 0.32 (p=0.169) | -0.06 (p=0.790) | -0.19 (p=0.416) | 0.22 (p=0.353) | 0.14 (p=0.564) | **0.55 (p=0.012)** | 0.21 (p=0.363) |
| **Evans Index** | -0.21 (p=0.366) | 0.36 (p=0.124) | -0.03 (p=0.891) | -0.15 (p=0.529) | 0.16 (p=0.508) | 0.09 (p=0.693) | **0.58 (p=0.007)** | 0.10 (p=0.660) |

**Abbreviations: NPi=** Neurological Pupil Index**, AP=** Anterior-Posterior

**Table 3.** Correlation of **Preoperative Right Sided Pupillometry** and Imaging Findings in Patients with Skull Base Tumors (n=37)

|  | **NPi** | **Size** | **Min. Size** | **Constr.** | **Constr. Velocity** | **Max Constr. Velocity** | **Latency** | **Dilatation Velocity** |
| --- | --- | --- | --- | --- | --- | --- | --- | --- |
| **Tumor Volume** | 0.19 (p=0.557) | -0.24 (p=0.457) | 0.34 (p=0.286) | 0.17 (p=0.602) | 0.02 (p=0.948) | -0.13 (p=0.697) | -0.09 (p=0.779) | 0.42 (p=0.175) |
| **4th Ventricle Volume** | 0.10 (p=0.762) | 0.30 (p=0.342) | -0.49 (p=0.106) | -0.33 (p=0.297) | -0.19 (p=0.557) | 0.45 (p=0.145) | -0.16 (p=0.618) | 0.04 (p=0.897) |
| **AP-Displacement** | -0.19 (p=0.555) | 0.23 (p=0.476) | 0.02 (p=0.957) | -0.17 (p=0.601) | -0.30 (p=0.346) | 0.04 (p=0.897) | **-0.67 (p=0.018)** | 0.36 (p=0.248) |
| **Midline Shift** | 0.03 (p=0.930) | -0.16 (p=0.628) | **0.76 (p=0.004)** | -0.01 (p=0.974) | 0.20 (p=0.543) | 0.18 (p=0.573) | -0.10 (p=0.750) | 0.04 (p=0.895) |
| **Diam. of Lat. Ventricles** | -0.39 (p=0.214) | -0.17 (p=0.599) | 0.27 (p=0.388) | 0.20 (p=0.524) | 0.04 (p=0.896) | -0.02 (p=0.948) | -0.26 (p=0.413) | 0.20 (p=0.524) |
| **Evans Index** | -0.27 (p=0.394) | -0.21 (p=0.519) | 0.22 (p=0.490) | 0.11 (p=0.741) | -0.07 (p=0.834) | -0.05 (p=0.878) | -0.32 (p=0.303) | 0.21 (p=0.504) |

**Abbreviations: NPi=** Neurological Pupil Index**, AP=** Anterior-Posterior

*Note: Spearman correlation coefficients (ρ) and corresponding unadjusted p-values are presented. A Benjamini–Hochberg false discovery rate correction was applied across all 288 correlation tests*

**Table 4.** Correlation of **Preoperative Left Sided Pupillometry** and Imaging Findings in Patients with Skull Base Tumors (n=37)

|  | **NPi** | **Size** | **Min. Size** | **Constr.** | **Constr. Velocity** | **Max Constr. Velocity** | **Latency** | **Dilatation Velocity** |
| --- | --- | --- | --- | --- | --- | --- | --- | --- |
| **Tumor Volume** | **-0.72 (p=0.008)** | 0.56 (p=0.059) | -0.49 (p=0.106) | 0.03 (p=0.914) | **0.73 (p=0.007)** | -0.29 (p=0.366) | 0.48 (p=0.112) | -0.16 (p=0.618) |
| **4th Ventricle Volume** | 0.38 (p=0.217) | -0.11 (p=0.729) | 0.04 (p=0.897) | -0.20 (p=0.542) | -0.32 (p=0.308) | 0.28 (p=0.379) | -0.16 (p=0.618) | -0.25 (p=0.430) |
| **AP-Displacement** | 0.01 (p=0.965) | -0.11 (p=0.736) | -0.01 (p=0.983) | -0.36 (p=0.253) | -0.00 (p=0.991) | -0.32 (p=0.317) | -0.02 (p=0.957) | 0.22 (p=0.490) |
| **Midline Shift** | -0.40 (p=0.196) | 0.26 (p=0.422) | 0.28 (p=0.371) | -0.34 (p=0.283) | **0.60 (p=0.040)** | -0.10 (p=0.750) | 0.40 (p=0.200) | -0.12 (p=0.700) |
| **Diam. of Lat. Ventricles** | 0.12 (p=0.711) | 0.32 (p=0.304) | 0.19 (p=0.554) | -0.18 (p=0.569) | 0.36 (p=0.252) | 0.32 (p=0.304) | 0.42 (p=0.179) | -0.13 (p=0.678) |
| **Evans Index** | -0.01 (p=0.965) | 0.37 (p=0.240) | 0.10 (p=0.749) | -0.20 (p=0.526) | 0.37 (p=0.231) | 0.32 (p=0.303) | **0.58 (p=0.046)** | -0.22 (p=0.490) |

**Abbreviations: NPi=** Neurological Pupil Index**, AP=** Anterior-Posterior

*Note: Spearman correlation coefficients (ρ) and corresponding unadjusted p-values are presented. A Benjamini–Hochberg false discovery rate correction was applied across all 288 correlation tests*

**Table 5. Pre- vs. Postop Pupillometry results of the metastasis group analyzed by Wilcoxon signed rank analysis**

| **Metric** | **n** | **Preop Median (IQR)** | **Postop Median (IQR)** | **Wilcoxon p-value** |
| --- | --- | --- | --- | --- |
| **NPi right** | 21 | 4.40 (3.90–4.50) | 4.40 (3.90–4.60) | 0.7932 |
| **Constriction Velocity (CV) (CV) right** | 17 | 2.01 (1.16–2.68) | 1.80 (1.29–1.88) | 0.1743 |
| **Minimal Pupil Size right** | 17 | 2.69 (2.31–3.17) | 2.60 (2.18–2.89) | 0.1454 |
| **NPi left** | 21 | 4.40 (4.00–4.60) | 4.40 (3.80–4.60) | 0.3693 |
| **Constriction Velocity (CV) (CV) left** | 17 | 2.06 (1.40–2.66) | 1.80 (1.22–2.31) | 0.4874 |
| **Minimal Pupil Size left** | 17 | 2.57 (2.16–2.99) | 2.36 (2.10–2.93) | 0.2247 |

**Abbreviations: NPi=** Neurological Pupil Index, **IQR=** Interquartile Range

*Note: Spearman correlation coefficients (ρ) and corresponding unadjusted p-values are presented. A Benjamini–Hochberg false discovery rate correction was applied across all 288 correlation tests*

**Table 6. Pre- vs. Postop Pupillometry results of the skull base group analyzed by Wilcoxon signed rank analysis**

| **Metric** | **n** | **Pre Median (IQR)** | **Post Median (IQR)** | **Wilcoxon p-value** |
| --- | --- | --- | --- | --- |
| **NPi right** | 35 | 4.40 (4.05–4.55) | 4.50 (4.05–4.70) | **0.0229** |
| **Constriction Velocity (CV) right** | 30 | 2.17 (1.40–2.56) | 2.34 (1.64–2.81) | 0.2801 |
| **Minimal Pupil Size right** | 30 | 2.75 (2.32–2.99) | 2.41 (2.14–2.80) | **0.0133** |
| **NPi left** | 35 | 4.40 (4.15–4.60) | 4.50 (4.20–4.65) | **0.0284** |
| **Constriction Velocity (CV) left** | 30 | 2.30 (1.76–2.72) | 2.34 (1.50–2.77) | 0.7922 |
| **Minimal Pupil Size left** | 30 | 2.65 (2.33–3.00) | 2.38 (2.15–2.77) | **0.0030** |

**Abbreviations: NPi=** Neurological Pupil Index, **IQR=** Interquartile Range

*Note: Spearman correlation coefficients (ρ) and corresponding unadjusted p-values are presented. A Benjamini–Hochberg false discovery rate correction was applied across all 288 correlation tests*

**Supplementary Table S7. Exploratory comparison of quantitative pupillometry in skull base meningiomas versus vestibular schwannomas**

*.*

| **Metric** | **Meningioma WHO grade 1 (n = 14)** | **Vestibular schwannoma / neurinoma WHO grade 1 (n = 9)** | **p-value*** |
| --- | --- | --- | --- |
| Preoperative NPi right | 4.3 (4.1–4.5, 2.7–4.7) | 4.4 (4.2–4.6, 3.6–4.7) | 0.727 |
| Preoperative NPi left | 4.4 (4.2–4.6, 2.7–4.7) | 4.4 (4.2–4.6, 3.6–4.7) | 0.874 |
| Postoperative NPi right | 4.6 (4.4–4.7, 3.3–4.8) | 4.6 (4.0–4.6, 3.9–4.8) | 0.787 |
| Postoperative NPi left | 4.6 (4.4–4.7, 3.7–4.8) | 4.6 (4.2–4.7, 3.9–4.8) | 1.000 |
| ΔNPi right (post–pre) | 0.2 (0.0–0.3, −0.1–0.9) | 0.1 (−0.1–0.3, −0.4–0.5) | 0.440 |
| ΔNPi left (post–pre) | 0.1 (0.0–0.3, −0.2–1.5) | 0.1 (0.1–0.3, −0.3–0.6) | 0.866 |

*Data are median (IQR, range). ΔNPi = postoperative – preoperative NPi. P-values from Mann–Whitney U tests* *Mann–Whitney U test.

*Note: Spearman correlation coefficients (ρ) and corresponding unadjusted p-values are presented. A Benjamini–Hochberg false discovery rate correction was applied across all 288 correlation tests*

***Supplementary Table 8. Correlations with unadjusted p < 0.05 and corresponding FDR-adjusted q-values (Benjamini–Hochberg across all 288 tests)***

| **Cohort & eye (original table)** | **Imaging variable** | **Pupillometry metric** | **ρ** | **p (uncorrected)** | **q (FDR-adjusted)** |
| --- | --- | --- | --- | --- | --- |
| Overall cohort – left eye (Table 6) | Tumor Volume | NPi | −0.54 | 0.013 | 0.37 |
| Overall cohort – left eye (Table 6) | Tumor Volume | Constriction Velocity (CV) | 0.53 | 0.015 | 0.37 |
| Overall cohort – left eye (Table 6) | Midline Shift | Constriction Velocity (CV) | 0.53 | 0.017 | 0.37 |
| Overall cohort – left eye (Table 6) | Diam. of Lateral Ventricles | Latency | 0.55 | 0.012 | 0.37 |
| Overall cohort – left eye (Table 6) | Evans Index | Latency | 0.58 | 0.007 | 0.37 |
| Metastasis – left eye (Table 8) | Tumor Volume | NPi | −0.54 | 0.013 | 0.37 |
| Metastasis – left eye (Table 8) | Tumor Volume | Constriction Velocity (CV) | 0.53 | 0.015 | 0.37 |
| Metastasis – left eye (Table 8) | Midline Shift | Constriction Velocity (CV) | 0.53 | 0.017 | 0.37 |
| Metastasis – left eye (Table 8) | Diam. of Lateral Ventricles | Latency | 0.55 | 0.012 | 0.37 |
| Metastasis – left eye (Table 8) | Evans Index | Latency | 0.58 | 0.007 | 0.37 |
| Skull base tumours – right eye (Table 9) | Midline Shift | Min. Pupil Size | 0.76 | 0.004 | 0.37 |
| Skull base tumours – right eye (Table 9) | AP-Displacement | Latency | −0.67 | 0.018 | 0.37 |
| Skull base tumours – left eye (Table 10) | Tumor Volume | NPi | −0.72 | 0.008 | 0.37 |
| Skull base tumours – left eye (Table 10) | Tumor Volume | Constriction Velocity (CV) | 0.73 | 0.007 | 0.37 |
| Skull base tumours – left eye (Table 10) | Midline Shift | Constriction Velocity (CV) | 0.60 | 0.040 | 0.77 |
| Skull base tumours – left eye (Table 10) | Evans Index | Latency | 0.58 | 0.046 | 0.83 |

**Supplementary Table S9 – Pre- and postoperative pupillometry according to radiological brainstem compression**

| **Pupillometry metric (eye)** | **No compression (n = 24) median (IQR)** | **Compression present (n = 34) median (IQR)** | **p-value (unadjusted)*** |
| --- | --- | --- | --- |
| **Pre-op NPi right** | 4.5 (4.3–4.6) | 4.2 (3.9–4.4) | 0.041 |
| **Pre-op NPi left** | 4.5 (4.2–4.6) | 4.1 (3.8–4.3) | 0.022 |
| **Post-op NPi right** | 4.6 (4.4–4.7) | 4.5 (4.2–4.7) | 0.331 |
| **Post-op NPi left** | 4.6 (4.4–4.7) | 4.5 (4.2–4.7) | 0.364 |
| **ΔNPi right (post–pre)** | +0.1 (0.0–0.3) | +0.3 (0.1–0.6) | 0.048 |
| **ΔNPi left (post–pre)** | +0.1 (0.0–0.3) | +0.2 (0.0–0.4) | 0.061 |
| **Pre-op Constr. velocity right (mm/s)** | 2.3 (2.0–2.5) | 2.0 (1.7–2.3) | 0.052 |
| **Pre-op Constr. velocity left (mm/s)** | 2.2 (2.0–2.4) | 1.9 (1.6–2.2) | 0.038 |
| **Pre-op Min. pupil size left (mm)** | 2.8 (2.5–3.1) | 3.2 (2.8–3.6) | 0.044 |

*Mann–Whitney U test, unadjusted; exploratory only.
**Abbreviations:** NPi = Neurological Pupil index; Constr. = Constriction; Min. = Minimal.
After false-discovery-rate correction across all exploratory tests, no correlation remained statistically significant.

**Supplementary Table S10 – Pre-operative pupillometry according to neurological signs at presentation**

| **Pupillometry metric (eye)** | **No neurological signs (n = 10) median (IQR)** | **≥ 1 neurological sign (n = 48) median (IQR)** | **p-value (unadjusted)*** |
| --- | --- | --- | --- |
| **Pre-op NPi right** | 4.5 (4.3–4.7) | 4.3 (4.0–4.5) | 0.180 |
| **Pre-op NPi left** | 4.5 (4.3–4.7) | 4.2 (3.9–4.4) | 0.132 |
| **Pre-op Constr. velocity right (mm/s)** | 2.3 (2.1–2.5) | 2.0 (1.8–2.3) | 0.089 |
| **Pre-op Constr. velocity left (mm/s)** | 2.2 (2.0–2.4) | 1.9 (1.7–2.2) | 0.077 |
| **Pre-op Min. pupil size left (mm)** | 2.9 (2.6–3.2) | 3.1 (2.7–3.5) | 0.156 |

*Mann–Whitney U test, unadjusted; exploratory only.
**Abbreviations:** NPi = Neurological Pupil index; Constr. = Constriction; Min. = Minimal.
